# Meta-analysis of the efficacy of Ningmitai capsule on the treatment of chronic prostatitis in China: Erratum

**DOI:** 10.1097/MD.0000000000012511

**Published:** 2018-09-14

In the article, “Meta-analysis of the efficacy of Ningmitai capsule on the treatment of chronic prostatitis in China”,^[1]^ which appeared Volume 97, Issue 33 of *Medicine*, all instances of NB in Figure 1 should be NMT. The corrected figure is below.

**Figure:**
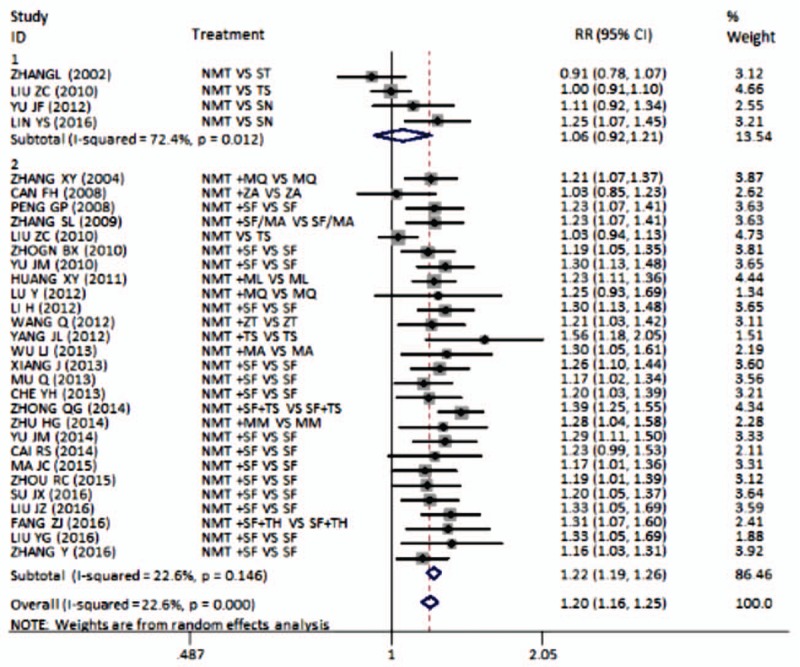


**Figure 1.** Meta-analysis on the effective rate of comparison of NMT with antibiotics. NMT = Ningmitai.
